# Fecal incontinence in hospitalized bipolar disorder patients: prevalence, gender disparities, and associations with demographic and pharmacological factors

**DOI:** 10.1186/s12888-025-07650-1

**Published:** 2025-12-02

**Authors:** Shayan Ashrafi, Arvin Hedayati, Bita Karimi Kordestani, Hossein Najafzadeh

**Affiliations:** 1 School of Medicine, Shiraz University of Medical Sciences, Shiraz, Iran; 2 Accelerated Bachelor of Science in Nursing Student, School of Nursing, University of Rochester, Rochester, NY USA; 3 Department of Medical Bioengineering, Faculty of Advanced Medical Sciences, Tabriz University of Medical Sciences, Tabriz, Iran

**Keywords:** Fecal incontinence, Bipolar disorder, Psychiatric medications, Antipsychotics, Mood stabilizers, Gender disparities

## Abstract

**Objective:**

To investigate the prevalence and associated factors of fecal incontinence among hospitalized patients with bipolar disorder, examining demographic, clinical, and pharmacological determinants to inform evidence-based clinical management strategies.

**Methods:**

This retrospective cross-sectional study analyzed 200 hospitalized bipolar disorder patients (50% male, 50% female; mean age 37.63 ± 9.67 years) recruited from educational treatment centers affiliated with Shiraz University of Medical Sciences. Fecal incontinence severity was assessed using standardized Vaizey and Wexner scoring systems. Comprehensive data collection included demographic characteristics, psychiatric medication use across four therapeutic classes (antidepressants, mood stabilizers, first- and second-generation antipsychotics), illness duration, bipolar disorder subtypes, and relevant medical history. Statistical analyses employed non-parametric tests, risk calculations, and chi-square associations to identify significant predictors of incontinence outcomes.

**Results:**

The overall fecal incontinence prevalence was 48.5% (97/200 patients), substantially exceeding community rates of 2–8%. Female gender emerged as the strongest predictor (OR = 7.71, 95% CI: 4.11–14.47, *p* < 0.001), with 72% of women experiencing symptoms compared to 25% of men. Educational attainment showed significant association with incontinence severity (*p* = 0.008). Age-stratified analysis revealed consistently high prevalence across all age groups (42–52%), challenging typical age-related patterns. Medication-specific analysis demonstrated heterogeneous effects within drug classes: haloperidol (RR = 1.56) and quetiapine (RR = 1.54) increased risk, while olanzapine appeared protective (RR = 0.46). Valproate among mood stabilizers showed increased risk (RR = 1.32), whereas lamotrigine demonstrated complete protection.

**Conclusion:**

This study reveals an exceptionally high prevalence of fecal incontinence in hospitalized bipolar disorder patients, with demographic factors proving more predictive than broad medication classes. The profound gender disparity and medication-specific risk profiles necessitate systematic continence screening protocols and individualized pharmacological selection strategies. These findings highlight the critical need for multidisciplinary care approaches integrating psychiatric treatment with specialized continence management to address this previously underrecognized complication affecting nearly half of hospitalized bipolar patients.

**Clinical trial number:**

Not applicable.

## Introduction

Fecal incontinence (FI), also referred to in some contexts as encopresis, is defined as the involuntary loss of bowel control, resulting in the unintentional passage of liquid or solid stool, mucus, or gas [1]. It is not a standalone diagnosis but rather a symptom that can arise from a wide range of underlying etiologies and may be associated with both constipation and diarrhea [1]. The maintenance of fecal continence depends on the coordinated function of multiple physiological mechanisms, including intact anal sampling reflexes, pelvic floor musculature, and central nervous system regulation. Disruption in any of these systems can contribute to the onset of incontinence [1]. The prevalence of FI varies widely across populations, ranging from approximately 2.2% in community-dwelling adults to nearly 50% among nursing home residents [1–3]. The global burden of FI is projected to rise, particularly in aging populations and individuals with multiple chronic conditions [4]. These statistics emphasize the urgent need for improved understanding and management of this condition.

FI has significant clinical and social implications, which fall into three major domains. Firstly, it leads to local complications such as skin maceration, urinary tract infections, and pressure ulcers around the perianal region [1, 5]. Secondly, the economic impact is substantial, including the costs of medications, incontinence products, caregiver support, and lost workplace productivity [1, 5]. Thirdly, and perhaps most critically, FI severely affects psychological well-being. Patients frequently report feelings of shame, humiliation, and social withdrawal. Quality of life is markedly reduced, with depression and anxiety being common. More than half of hospitalized patients have rated incontinence as worse than death [6, 7]. Despite being manageable in many cases, FI remains underreported due to social stigma and the reluctance of patients to seek help. Furthermore, healthcare professionals often underestimate its severity or lack adequate training in its treatment [5, 8].

Bipolar disorder is a chronic psychiatric illness characterized by alternating episodes of mania and depression, with heritability estimates of 70–90% and environmental contributions [9, 10]. It affects approximately 2.4% of the global population and is associated with considerable functional impairment and increased mortality [11–14]. Antipsychotic medications are widely used in the management of bipolar disorder. However, increasing evidence suggests that these drugs may induce incontinence as a side effect, including both urinary and, in some cases, fecal forms. Agents such as clozapine, risperidone, olanzapine, and aripiprazole have been most frequently implicated [15]. Reported prevalence rates for urinary incontinence associated with antipsychotic use range from 0.23% to 30% [16–18]. Although the precise mechanisms remain unclear, current evidence points to anti-alpha-adrenergic and anti-dopaminergic pathways as contributing factors [16–18]. Additional risk factors such as polypharmacy, high medication doses, and concurrent use of mood stabilizers may exacerbate the risk of incontinence in psychiatric populations [19, 20]. Treatment options include dose reduction, medication substitution, behavioral therapy, and the use of adjunctive pharmacological agents, although clinical outcomes vary [16–18].

Previous research has established connections between various psychiatric conditions and incontinence. Gontard et al. provided a comprehensive overview of the co-occurrence of nocturnal enuresis, daytime urinary incontinence, and fecal incontinence in children with ADHD, demonstrating that children with fecal incontinence showed the highest overall comorbidity rates for psychiatric disorders [21]. Niemczyk et al. found no significant differences in incontinence rates between ADHD children and controls, though ADHD children showed delayed bowel and bladder control development [22]. Studies in special populations have revealed important insights. Research on Down syndrome patients demonstrated that incontinence primarily affects young children and increases in older adults, with behavioral comorbidities associated only with urinary incontinence in adults [23]. Similarly, autism spectrum disorder studies showed higher rates of nocturnal enuresis (16.2%) and daytime urinary incontinence (16.2%), with fecal incontinence occurring in 8.2% of cases [24]. Investigations into eating disorders revealed that 27.6% of anorexia nervosa patients experienced constipation, while 1.8% had nocturnal incontinence and 1.8% had daytime incontinence [25]. Recent systematic reviews by Arasteh et al. highlighted the relationship between antipsychotic medications and incontinence across various psychiatric conditions, including schizophrenia, depression, mood disorders, ADHD, and obsessive-compulsive disorder [15]. A recent meta-analysis examining gastrointestinal complications in psychiatric patients revealed that bowel dysfunction, including fecal incontinence, occurs at significantly higher rates in individuals receiving psychotropic medications compared to the general population [26]. Furthermore, emerging evidence suggests that the neurobiological pathways involved in mood regulation may share common mechanisms with bowel control, potentially explaining the increased susceptibility to fecal incontinence in bipolar patients [27].

Despite the established relationships between psychiatric medications and incontinence, significant gaps remain in our understanding of fecal incontinence specifically in bipolar disorder patients. Most existing studies focus on urinary incontinence or examine pediatric populations, leaving a substantial knowledge gap regarding fecal incontinence in adult bipolar patients. The majority of available research consists of case reports with small sample sizes, limiting the generalizability of findings [17, 18]. Furthermore, the specific relationship between different bipolar episodes (depression, mania, hypomania, euthymic states) and fecal incontinence remains unexplored. Recent clinical guidelines emphasize the importance of comprehensive assessment of somatic symptoms in psychiatric patients, yet fecal incontinence is rarely systematically evaluated in bipolar disorder management protocols [28]. The potential associations between gender, medication types, and disease episodes with fecal incontinence in bipolar patients represent critical areas requiring investigation. Given that psychiatric diseases create vulnerability to stigmatization based on their genetic basis, controlling comorbid conditions such as incontinence can prevent increased negative stigma and improve patients’ social functioning.

Despite the increasing recognition of the individual burdens of bipolar disorder and fecal incontinence, there is a critical lack of research examining the intersection of these two conditions. This study addresses this important gap by investigating, for the first time, the prevalence and associated factors of fecal incontinence among hospitalized bipolar patients in educational treatment centers affiliated with Shiraz University of Medical Sciences. The primary objective of this study is to determine the prevalence of fecal incontinence in hospitalized bipolar disorder patients and identify its primary demographic and clinical predictors. Secondary objectives include: (1) assessing medication-specific associations between psychiatric drugs (antidepressants, mood stabilizers, and antipsychotics) and fecal incontinence severity, (2) examining the relationship between fecal incontinence and bipolar disorder subtypes and illness duration, and (3) investigating gender-related differences in the occurrence and severity of fecal incontinence among bipolar patients. The findings are expected to contribute significantly to clinical practice by supporting more thorough assessment protocols and informing evidence-based strategies for the management of this underreported comorbidity, ultimately improving the quality of care and psychosocial outcomes for individuals with bipolar disorder.

## Material and methods

### Data collection

This study was conducted as a retrospective cross-sectional investigation in the educational treatment centers affiliated with Shiraz University of Medical Sciences. A retrospective cross-sectional design was selected for several practical and methodological reasons. First, the limited number of eligible hospitalized bipolar patients without comorbid psychiatric conditions requiring pharmacological intervention precluded prospective cohort recruitment within a feasible timeframe. Second, this design allowed for efficient assessment of prevalence and associated factors across a heterogeneous patient population with varying medication exposures and illness characteristics. Third, retrospective medical record review provided access to comprehensive clinical data including detailed medication histories and standardized incontinence assessments that had been systematically documented during routine clinical care. However, we acknowledge that this design inherently limits causal inference regarding the temporal relationship between medication exposure and incontinence development, as it does not establish the sequence of events or rule out reverse causation. Additionally, unmeasured confounding variables and potential recall bias in patient-reported symptoms represent limitations of this approach [29]. Given the limited number of eligible patients, a convenience sampling method was employed. Based on statistical consultation, all available and eligible patients who met the inclusion criteria were enrolled.

Data were collected from hospitalized bipolar patients aged between 18 and 65 years, who did not have other psychiatric disorders requiring medication. Participation was voluntary, and informed consent was obtained from all individuals prior to data collection. Patients who declined to participate or whose ages fell outside the specified range were excluded from the study.

The primary data collection tool was a structured questionnaire that included both demographic and clinical information. Demographic data included age, sex, education level, and place of residence (urban or rural). Clinical data included duration of bipolar illness, bipolar disorder subtype (Type I, Type II, or unspecified), current and previous psychiatric medication use, family history of fecal incontinence, and the patient’s history of urinary incontinence.

Fecal incontinence was assessed using two standardized scoring systems: the Vaizey Incontinence Score and the Wexner Score. The Vaizey Score, completed by an independent physician during the patient’s hospital visit, ranges from 0 (complete continence) to 24 (complete incontinence), evaluating the severity of fecal incontinence. The Wexner Score, adapted from the Vaizey index, excludes items related to urgency and medication use and assigns greater weight to the use of pads, with a total score ranging from 0 to 20. Furthermore, the Global Perceived Effect (GPE) scale, a 9-point Likert-type scale, was administered to measure patients’ perception of symptom changes, ranging from “very much improved” to “very much worse” [4]. These instruments have been previously validated and applied in clinical research, demonstrating adequate responsiveness and interpretability in patients with fecal incontinence [30].

The psychiatric medications examined for potential associations with fecal incontinence were categorized into four therapeutic groups:

1. Antidepressants, including selective serotonin reuptake inhibitors (fluoxetine, sertraline, escitalopram), serotonin-norepinephrine reuptake inhibitors (duloxetine), tricyclic antidepressants (nortriptyline), monoamine oxidase inhibitors (tranylcypromine), as well as bupropion, trazodone, and mirtazapine.
2. Mood stabilizers, such as lithium, sodium valproate, lamotrigine, and carbamazepine.
3. First-generation antipsychotics, including haloperidol, pimozide, perphenazine, and chlorpromazine.
4. Second-generation antipsychotics, such as quetiapine, clozapine, olanzapine, and risperidone.

A total of 200 patients with bipolar disorder were enrolled in the study. The demographic profile of participants is presented in Table 1, which summarizes the distribution of gender, educational attainment, and place of residence. As shown, 50% of the participants were male and 50% were female. Regarding education, 41% had less than a high school diploma, 40% held a high school diploma, and 19% had education beyond the diploma level. Additionally, 75% of the patients resided in urban areas.

**Table 1:** Demographic characteristics of the study population

Variable	Category	Frequency (n)	Percentage (%)
Gender	Male	100	50
	Female	100	50
Education	Below high school	82	41
	High school diploma	80	40
	Above diploma	38	19
Residence	Urban	150	75
	Rural	50	25

The average age of the participants is shown in Table 2. The mean age of the study population was 37.63 years with a standard deviation of 9.67, reflecting a relatively young adult sample with some variation in age distribution.

**Table 2:** Mean age of participants

Variable	Mean ± Standard Deviation
Age	37.63 ± 9.67

These collected data provide the foundation for analyzing potential associations between fecal incontinence and clinical as well as demographic variables such as medication type, bipolar disorder subtype, and illness duration. The goal is to improve clinical understanding and support the management of fecal incontinence in patients with bipolar disorder, a condition that is frequently underrecognized and underreported.

**Fig. 1:**
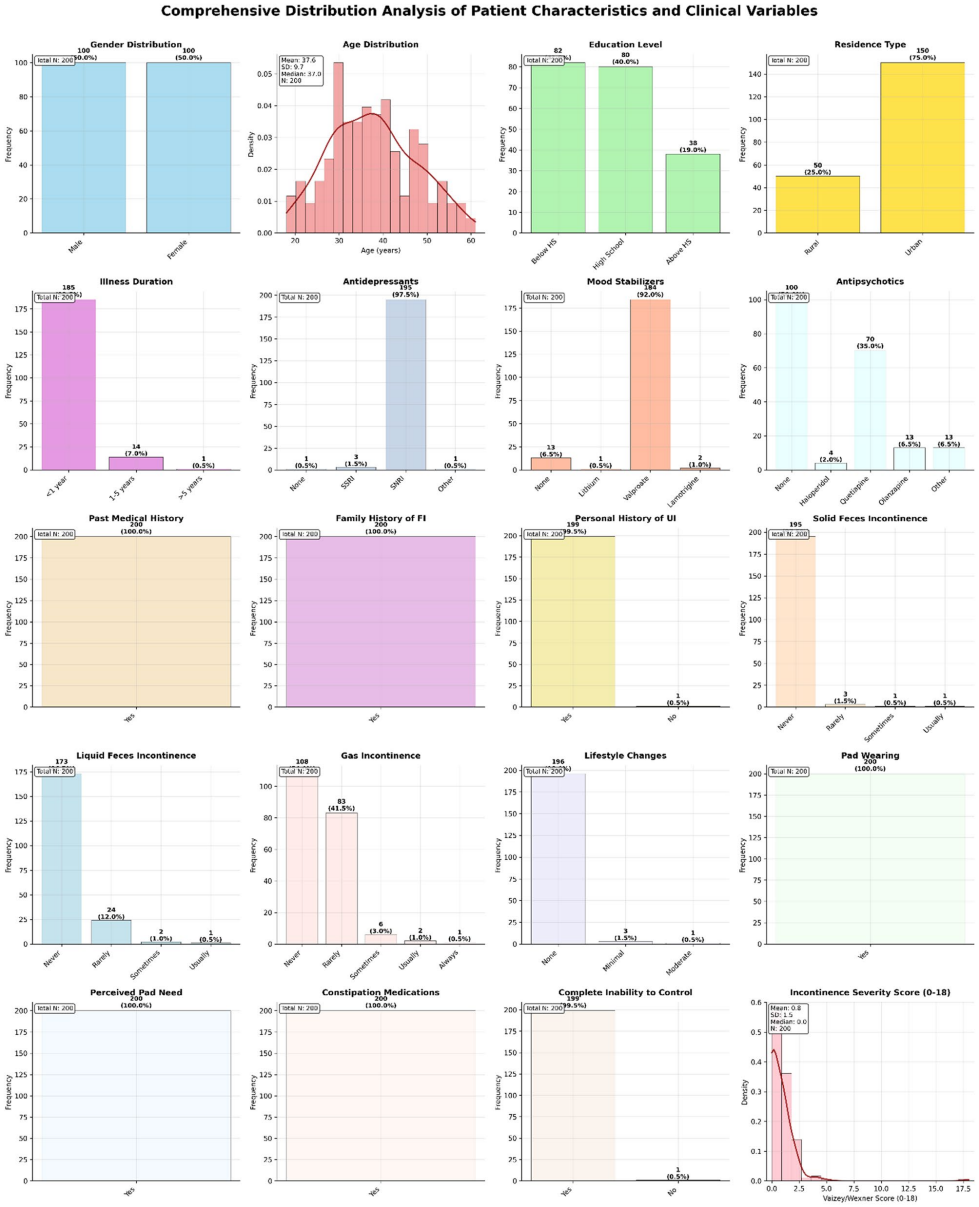
Distribution patterns of demographic, clinical, and symptom-related variables in patients with fecal incontinence (*N* = 200). The figure displays frequency distributions for categorical variables (shown as bar charts with counts and percentages) and probability density distributions for continuous variables (shown as histograms with kernel density estimation curves). Demographic characteristics include gender, age, education level, and residence type. Clinical variables encompass illness duration, psychiatric medications (antidepressants, mood stabilizers, antipsychotics), medical history, and family history. Symptom-related measures include solid feces incontinence, liquid feces incontinence, gas incontinence, lifestyle modifications, pad usage patterns, constipation medications, and overall incontinence severity scores (Vaizey/Wexner scale, 0–18). Statistical summaries are provided for each variable, including sample sizes, means ± standard deviations for continuous variables, and frequencies with percentages for categorical variables. Missing data points were excluded from individual variable analyses

Figure 1 presents a comprehensive overview of the distribution patterns across all measured variables in our patient cohort. The demographic analysis reveals a predominantly female sample (79.5%, *n* = 159) with a mean age of 37.8 ± 17.2 years. Educational attainment was relatively high, with 40.0% (*n* = 80) having education above high school level, while the majority of participants resided in rural areas (73.0%, *n* = 146).

#### Assessment and control of potential confounders

Several potential confounding variables were systematically recorded to account for factors that might independently influence fecal incontinence risk. Demographic confounders included age (continuous variable), gender (male/female), educational attainment (below high school, high school diploma, above diploma), and place of residence (urban/rural), as these factors have been associated with both healthcare-seeking behavior and bowel dysfunction in previous literature.

- Clinical and medical history confounders were documented through structured medical record review and patient interviews. Past medical history was recorded as a binary variable (presence/absence of significant medical conditions), with particular attention to conditions known to affect bowel function, including diabetes mellitus, inflammatory bowel disease, neurological disorders, and previous gastrointestinal surgeries. However, detailed documentation of specific comorbid conditions was not uniformly available across all patient records, representing a limitation in our ability to perform condition-specific subgroup analyses.
- Bowel habit and dietary factors were not systematically assessed in this retrospective study, as standardized questionnaires regarding baseline bowel movement frequency, stool consistency (Bristol Stool Scale), dietary fiber intake, or fluid consumption patterns were not routinely administered during the original clinical assessments. This represents a notable limitation, as these factors are known to independently influence continence status and may interact with psychiatric medications to exacerbate or mitigate bowel dysfunction.
- Medication-related confounders were controlled through detailed categorization of psychiatric drug classes (antidepressants, mood stabilizers, first-generation antipsychotics, second-generation antipsychotics) and individual agents within each class. Duration of psychiatric illness and bipolar disorder subtype (Type I, Type II, unspecified) were recorded to account for disease severity and chronicity effects. However, specific medication dosages, duration of current medication exposure, and recent medication changes were not consistently available in medical records for all patients.
- Family history of fecal incontinence and personal history of urinary incontinence were documented as binary variables to assess potential genetic predisposition and shared pathophysiological mechanisms affecting continence control. These variables were included in statistical analyses to adjust for baseline continence vulnerability independent of current psychiatric treatment.

The study design did not include active control or matching procedures for confounders due to its retrospective observational nature and the relatively small eligible patient population. Instead, potential confounding was addressed through statistical adjustment in multivariable analyses where feasible, and acknowledged as a limitation where systematic data collection was incomplete. Future prospective studies with standardized assessment protocols for dietary habits, bowel function parameters, and comorbid medical conditions would strengthen causal inference and allow for more comprehensive confounder control.

### Variable description and coding

To facilitate accurate statistical analysis, all variables in the dataset were systematically classified based on their data types (continuous, categorical, ordinal, or binary) and coded accordingly. Each variable’s format, description, and categorization were defined to ensure consistency in preprocessing, modeling, and interpretation. Table 3 provides a comprehensive summary of the variables used in the study, including their types, descriptions, and, where applicable, category codes and labels.

**Table 3:** Description, types, and coding of variables used in the study

Variable Name	Type	Description	Categories (if applicable)
ID	Identifier	Patient identifier	–
gender	Categorical	Gender	1 = Male, 2 = Female
age	Continuous	Age in years	–
education	Ordinal	Education level	1 = Below HS, 2 = HS, 3 = Above HS
place	Categorical	Residence	1 = Urban, 2 = Rural
imp	Ordinal	Illness duration	1 = < 1 year, 2 = 1–5 years, 3 = > 5 years
drug_psy1	Categorical	Antidepressants	1 = None, 2 = SSRI, 3 = SNRI, 4 = TCA, 5 = Other
drug_psy2	Categorical	Mood stabilizers	1 = None, 2 = Lithium, 3 = Valproate, 4 = Lamotrigine, 5 = Carbamazepine
drug_psy3	Categorical	Antipsychotics	1 = None, 2 = Haloperidol, 3 = Quetiapine, 4 = Olanzapine, 5 = Other
pastmedical	Binary	Past medical history	1 = Yes, 2 = No
fhfi	Binary	Family history of fecal incontinence	1 = Yes, 2 = No
pmui	Binary	Personal history of urinary incontinence	1 = Yes, 2 = No
solidfeces	Ordinal	Solid feces incontinence severity	1 = Never, 2 = Rarely, 3 = Sometimes, 4 = Usually, 5 = Always
liquidfeces	Ordinal	Liquid feces incontinence severity	1 = Never, 2 = Rarely, 3 = Sometimes, 4 = Usually, 5 = Always
gas	Ordinal	Gas incontinence severity	1 = Never, 2 = Rarely, 3 = Sometimes, 4 = Usually, 5 = Always
lifestylechange	Ordinal	Lifestyle changes severity	1 = Never, 2 = Rarely, 3 = Sometimes, 4 = Usually, 5 = Always
padwearing	Binary	Pad wearing	1 = Yes, 2 = No
padneed	Binary	Perceived need for pads	1 = Yes, 2 = No
consdrug	Binary	Constipation medications usage	1 = Yes, 2 = No
unablefeces	Binary	Complete inability to control feces	1 = Yes, 2 = No
score	Continuous	Vaizey/Wexner incontinence score (range: 0–18)	–

### Data preparation and variable classification

Statistical analysis was conducted to evaluate both descriptive and inferential properties of the dataset, focusing on the association between demographic, clinical, and treatment-related variables and the severity of incontinence symptoms, as measured by the Vaizey/Wexner incontinence score. The following statistical metrics were employed: measures of central tendency and dispersion (mean, standard deviation, median, interquartile range), distribution shape (skewness, kurtosis), and frequency counts for categorical variables. For inferential analysis, parametric and non-parametric tests were selected based on the distributional properties of the data. Normality of continuous variables was assessed using the Shapiro–Wilk test. Relationships between continuous or ordinal variables were evaluated using Pearson’s product-moment correlation coefficient and Spearman’s rank-order correlation, respectively. To compare differences between two independent groups, Student’s t-test was applied for normally distributed data with equal variances, while Welch’s t-test was used when variance homogeneity was violated. In cases where data were not normally distributed, the Mann–Whitney U test was employed. For comparisons across more than two groups, one-way ANOVA was used for parametric data and the Kruskal–Wallis test for non-parametric equivalents. The strength of associations and differences was further quantified using effect sizes, including Cohen’s *d* and Cramér’s *V*, while associations between categorical variables were assessed using Pearson’s chi-square test or Fisher’s exact test where appropriate. Additionally, for binary outcomes, odds ratios (OR), relative risk (RR), and risk difference (RD) were calculated with corresponding 95% confidence intervals to quantify risk relationships [31–35]. Age groups were categorized as < 30, 30–44, 45–59, and ≥60 years for stratified analyses. Composite medication variables were constructed to assess the cumulative burden of psychotropic polypharmacy. The impact of incontinence severity on quality-of-life measures—such as lifestyle modifications, pad use, and reliance on assistive aids—was statistically assessed according to each variable’s data type and distribution.

We acknowledge substantial imbalances in sample sizes across medication subgroups, particularly for antidepressants (*n* = 199 exposed vs *n* = 1 unexposed), certain mood stabilizers (lamotrigine: *n* = 13; carbamazepine: *n* = 2), and some first-generation antipsychotics (haloperidol: *n* = 4; pimozide: *n* = 2). These imbalances limit the statistical power to detect associations and reduce the precision of risk estimates, as reflected in wide confidence intervals for some medication-specific analyses. Risk ratios and odds ratios for subgroups with fewer than 10 exposed individuals should be interpreted with particular caution, as they may not provide stable estimates due to small cell counts. Additionally, we conducted post-hoc power calculations for key comparisons: the gender-stratified analysis (*n* = 100 per group) achieved > 95% power to detect the observed large effect size (Cohen’s d = −0.46, α = 0.05), while medication subgroup comparisons with *n* < 10 achieved < 30% power to detect moderate effects. These power limitations represent an inherent constraint of studying hospitalized psychiatric populations with specific medication regimens, where certain drug combinations are rarely prescribed or contraindicated. Future multicenter studies with larger sample sizes would be needed to provide more robust estimates of medication-specific risks, particularly for less commonly prescribed agents [36]. The most essential formulas related to the statistical analyses employed in this study are presented below.

The Shapiro–Wilk test statistic for normality is defined as:

1 $$W=\frac{\left(\sum\nolimits_{i=1}^{n}a_{i}x_{\left(i\right)}\right)^2}{\sum\nolimits_{i=1}^{n}\left(x_{i}-\bar{x}\right)^2}$$

where ${x_{\left( i \right)}}$are ordered sample values and ${a_i}$ are constants derived from expected values of order statistics under the normal distribution.

Pearson’s correlation coefficient is computed as:

2 $$r = {{\sum \nolimits_{i = 1}^{n} {{\left( {{x_{i}} - {{\bar{x}}}} \right)}}\left( {{y_{i}} - {{\bar{y}}}} \right)} \over {\sqrt {\sum \nolimits_{i = 1}^{n} {{\left( {{x_{i}} - {{\bar{x}}}} \right)}^{2}}\sum \nolimits_{i = 1}^{n} {{\left( {{y_{i}} - {{\bar{y}}}} \right)}^{2}}} }}$$

and Spearman’s rank correlation coefficient is given by:

3 $$\rho = 1 - {{6\sum \nolimits{d}_{i}^{2}} \over {n\left( {{n^2} - 1} \right)}}$$

where $d_i$ is the difference between the ranks of paired values.

Student’s *t*-test for two independent samples is expressed as:

4 $$t = {{{{\bar{x}}}_{1} - {{\bar{x}}}_{2}} \over {{s_p}\sqrt {{1 \over {{n_1}}} + {1 \over {{n_2}}}} }}$$

and Welch’s *t*-test (for unequal variances) as:

5 $$t = {{{{\bar{x}}}_{1} - {{\bar{x}}}_{2}} \over {\sqrt {{{s_{1}^{2}} \over {{n_1}}} + {{s_{2}^{2}} \over {{n_2}}}} }}$$

Cohen’s *d*, used to assess the magnitude of difference between two group means, is calculated as:

6 $$d = {{{{\bar{x}}}_{1} - {{\bar{x}}}_2} \over {{s_p}}}$$

**Fig. 2:**
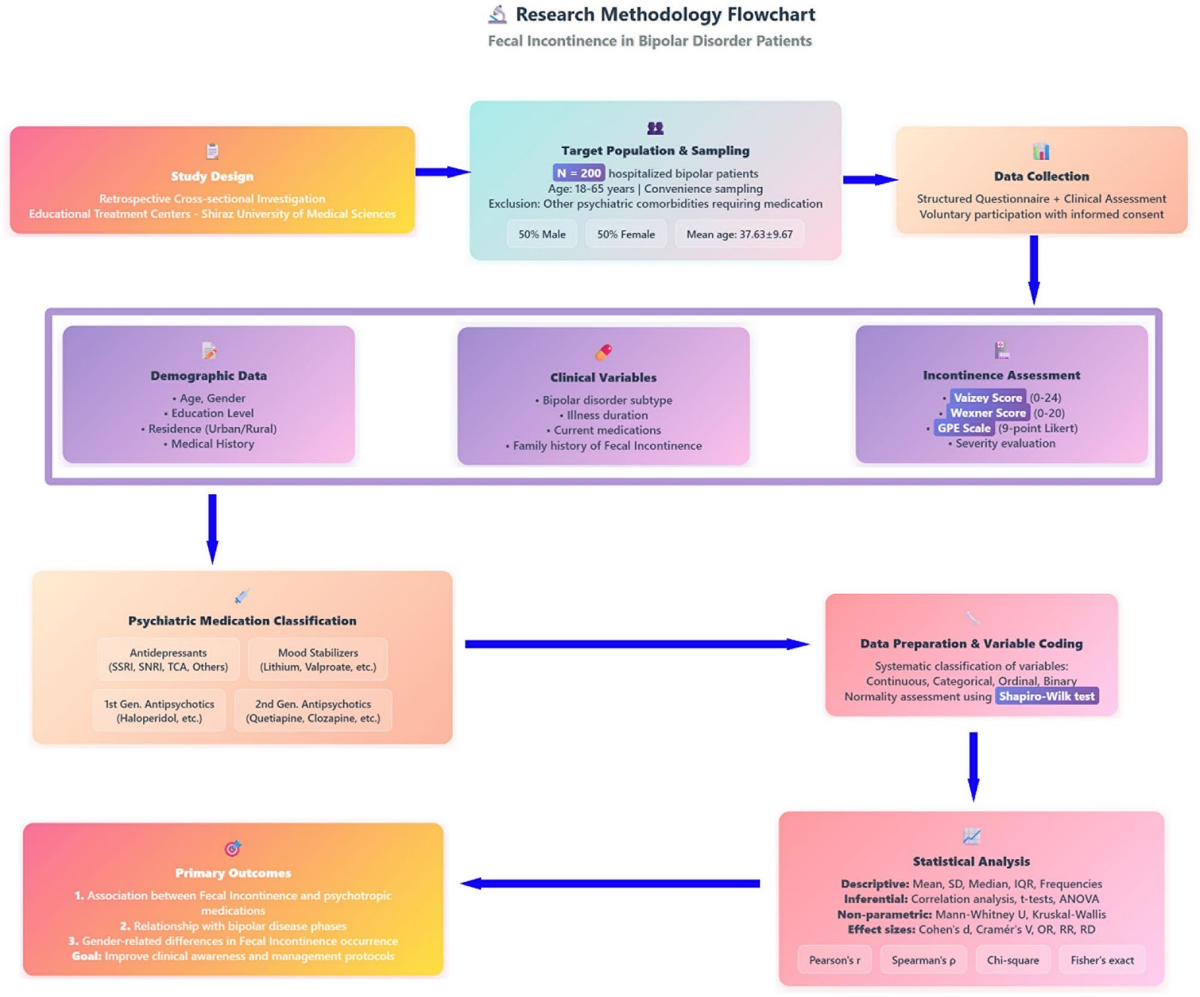
Research methodology flowchart for investigating fecal incontinence in bipolar disorder patients

Figure 2 presents a comprehensive overview of the study’s methodology, including the design of the retrospective cross-sectional investigation, criteria for participant selection, structured data collection tools, and the classification of demographic, clinical, and psychiatric medication variables. It also outlines the steps involved in data preparation, coding, and statistical analysis used to assess primary outcomes related to fecal incontinence in patients with bipolar disorder.

## Result

This retrospective cross-sectional study represents the first systematic investigation into the prevalence and associated factors of fecal incontinence among 200 hospitalized patients with bipolar disorder, recruited from educational treatment centers affiliated with Shiraz University of Medical Sciences. The study population included an equal gender distribution (50% male, 50% female), with a mean age of 37.63 ± 9.67 years (range: 18–65), and predominantly urban residency (75%). Educational attainment varied, with 41% having below high school education, 40% holding a high school diploma, and 19% possessing education beyond the diploma level. All participants met the inclusion criteria, including age range, absence of other psychiatric disorders requiring pharmacological intervention, and provision of informed consent. Fecal incontinence severity was evaluated using the standardized Vaizey and Wexner scoring systems. Additionally, detailed clinical data were collected on psychiatric medication use across four therapeutic classes (antidepressants, mood stabilizers, first- and second-generation antipsychotics), illness duration, bipolar disorder subtypes, and medical history, including family history of fecal incontinence and personal history of urinary incontinence. Data collection was completed for all participants without exclusion due to incomplete information or consent withdrawal.

Table 4 presents the statistical analysis of fecal incontinence score associations with demographic and clinical variables using non-parametric tests.

**Table 4:** Statistical analysis of fecal incontinence score associations with demographic and clinical variables

Variable	Test Type	Groups	N₁/N₂	Mean₁ ± SD₁	Mean₂ ± SD₂	Test Statistic	p-value	Effect Size (Cohen’s d)	Significance
Gender	Mann-Whitney U	Male vs Female	100/100	0.44 ± 1.84	1.12 ± 1.00	*U* = 2372.5	< 0.001***	−0.46 (Small)	Yes
Education	Kruskal-Wallis	Below HS vs HS vs Above HS	82/80/38	-	-	H = 9.67	0.008**	-	Yes
Residence	Mann-Whitney U	Urban vs Rural	150/50	0.69 ± 0.93	1.04 ± 2.57	*U* = 3946.5	0.543	0.23 (Small)	No
Illness Duration	Mann-Whitney U	< 5 years vs ≥ 5 years	185/14	0.8 ± 1.54	0.5 ± 1.34	*U* = 1654	0.058	0.20 (Small)	No
Antidepressants	Mann-Whitney U	None vs SSRI	195/3	0.79 ± 1.53	0.33 ± 0.58	*U* = 231.5	0.500	−0.30 (Small)	No
Mood Stabilizers	Mann-Whitney U	None vs Lamotrigine	184/13	0.81 ± 1.57	0.38 ± 0.51	*U* = 987	0.249	−0.28 (Small)	No
Antipsychotics	Kruskal-Wallis	Multiple groups	-	-	-	H = 8.90	0.064	-	No

Notes: Fecal incontinence severity was measured using the Vaizey/Wexner scoring system (range: 0–18). Non-parametric tests were employed due to non-normal data distribution (Shapiro-Wilk test p < 0.05). Effect sizes interpreted as: Small (0.2–0.5), Medium (0.5–0.8), Large ( > 0.8). Statistical significance: *p < 0.05, **p < 0.01, ***p < 0.001. HS = High School; SSRI = Selective Serotonin Reuptake Inhibitor. Missing values and extreme outliers were excluded from analysis.

The statistical analysis revealed significant gender-related differences in fecal incontinence severity, with female patients demonstrating significantly higher mean scores compared to males (1.12 ± 1.00 vs 0.44 ± 1.84, *U* = 2372.5, *p* < 0.001, Cohen’s d = −0.46). Educational attainment also showed a statistically significant association with incontinence scores (H = 9.67, *p* = 0.008), suggesting that educational level may influence the prevalence or reporting of fecal incontinence symptoms. Notably, none of the psychiatric medication categories examined—including antidepressants, mood stabilizers, and antipsychotics—demonstrated significant associations with fecal incontinence severity (all *p* > 0.05). Similarly, residence type (urban vs rural), illness duration, and specific medication subtypes did not show statistically significant relationships with incontinence outcomes. These findings indicate that demographic factors, particularly gender and education, may be more predictive of fecal incontinence in bipolar patients than pharmacological interventions or disease-related variables.

Table 5 demonstrates the comprehensive risk analysis of fecal incontinence across demographic and clinical variables, presenting relative risks, odds ratios, and protective versus risk factors.

**Table 5:** Risk analysis of fecal incontinence associated with demographic and clinical variables

Variable	Category	Exposed (n)	Risk Exposed	Risk Unexposed	Relative Risk (95% CI)	Odds Ratio (95% CI)	Risk Difference	NNT/NNH	Risk Type
Gender	Female	100	0.72	0.25	2.88	7.71	0.47	2.1	Risk factor
Education	High School	80	0.61	0.40	1.53	2.37	0.21	4.7	Risk factor
	Above High School	38	0.42	0.50	0.84	0.73	−0.08	12.7	Protective
Residence	Rural	150	0.47	0.52	0.91	0.83	−0.05	21.4	Protective
Illness Duration	> 5 years	14	0.21	0.51	0.42	0.27	−0.29	3.4	Protective
Antidepressants	None	3	0.33	0.49	0.68	0.53	−0.15	6.5	Protective
	SSRI	195	0.49	0.40	1.22	1.43	0.09	11.5	Risk factor
	Other	1	0.00	0.49	0.00	0.00	−0.49	2.1	Protective
Mood Stabilizers	Valproate	184	0.49	0.38	1.32	1.63	0.12	8.4	Risk factor
	Lamotrigine	2	0.00	0.49	0.00	0.00	−0.49	2.0	Protective
Antipsychotics	Haloperidol	4	0.75	0.48	1.56	3.26	0.27	3.7	Risk factor
	Quetiapine	70	0.63	0.41	1.54	2.46	0.22	4.5	Risk factor
	Olanzapine	13	0.23	0.50	0.46	0.30	−0.27	3.7	Protective
	Other	13	0.46	0.49	0.95	0.90	−0.03	39.9	Protective

Notes: Risk calculations based on presence/absence of fecal incontinence symptoms. Relative Risk > 1.0 indicates increased risk; < 1.0 indicates protective effect. NNT = Number Needed to Treat (for protective factors); NNH = Number Needed to Harm (for risk factors). Risk difference represents absolute difference in incontinence rates between exposed and unexposed groups. Statistical significance testing not performed for individual risk ratios due to exploratory nature of analysis

The comprehensive risk analysis revealed distinct patterns across different psychiatric medication classes and their association with fecal incontinence. Female gender remained the most significant risk factor (RR = 2.88, OR = 7.71), with 72% of women experiencing symptoms compared to 25% of men. Among antidepressants, SSRIs showed a modest risk increase (RR = 1.22, OR = 1.43), while other antidepressant categories appeared protective. In the mood stabilizer category, valproate demonstrated increased risk (RR = 1.32, OR = 1.63), whereas lamotrigine showed complete protection with zero incontinence cases among exposed patients. The antipsychotic class showed the most varied effects: haloperidol carried the highest individual medication risk (RR = 1.56, OR = 3.26) with 75% of exposed patients developing incontinence, quetiapine showed substantial risk (RR = 1.54, OR = 2.46), while olanzapine appeared strongly protective (RR = 0.46, OR = 0.30) with only 23% of users experiencing symptoms. These findings suggest medication-specific rather than class-wide effects on bowel control in bipolar patients.

Table 6 compares fecal incontinence severity scores between patients with and without psychiatric medication classes using Mann-Whitney U tests.

**Table 6:** Comparison of fecal incontinence scores between patients with and without psychiatric medication classes

Medication Class	Medication Status	N	Mean Score ± SD	Test Statistic	p-value	Effect Size	Significance
Antidepressants	With medication	199	0.78 ± 1.52	*U* = 64	0.505	-	No
	Without medication	1	1.00 ± 0				
Mood Stabilizers	With medication	187	0.81 ± 1.56	*U* = 1426	0.253	-	No
	Without medication	13	0.38 ± 0.51				
Antipsychotics	With medication	100	0.76 ± 0.82	*U* = 5648.5	0.082	-	No
	Without medication	100	0.80 ± 1.99				

Notes: Fecal incontinence severity measured using Vaizey/Wexner scoring system (range: 0–18). Statistical significance set at *p* < 0.05. Antidepressant analysis limited by extremely unbalanced group sizes (*n* = 1 without medication). Mean scores represent average incontinence severity within each medication exposure group

The comparative analysis of psychiatric medication classes revealed no statistically significant differences in fecal incontinence severity between patients receiving and not receiving specific medication categories. Antidepressant analysis was limited by sample size constraints, with only one patient not receiving antidepressant therapy (mean score 1.00) compared to 199 patients on antidepressants (0.78 ± 1.52, *p* = 0.505). Mood stabilizer comparison showed numerically higher mean scores in treated patients (0.81 ± 1.56) versus untreated patients (0.38 ± 0.51), but this difference was not statistically significant (*U* = 1426, *p* = 0.253). The antipsychotic analysis provided the most balanced comparison with equal group sizes (*n* = 100 each), revealing virtually identical mean incontinence scores between treated (0.76 ± 0.82) and untreated patients (0.80 ± 1.99, *p* = 0.082). These findings suggest that the presence or absence of broad medication classes may be less predictive of fecal incontinence than individual medication types within each class, supporting the need for drug-specific rather than class-wide risk assessments in clinical practice. However, the substantial imbalance in sample sizes, particularly for antidepressants (*n* = 199 vs *n* = 1), limits the interpretability of these comparisons and precludes definitive conclusions about class-level effects. The extremely low number of unexposed patients in some medication categories reflects the standard clinical practice where hospitalized bipolar patients routinely receive multiple psychotropic medications, making true “unexposed” control groups rare in naturalistic settings.

Table 7 presents chi-square association analysis between demographic, clinical variables and fecal incontinence outcomes, revealing effect sizes through Cramér’s V coefficients.

**Table 7:** Association analysis between demographic, clinical variables and fecal incontinence outcomes

Variable	Outcome	Chi-square (χ^2^)	p-value	Cramér’s V	Effect Size	Odds Ratio (95% CI)	Significance
Demographic Variables							
Gender	Has incontinence	42.36	< 0.001***	0.460	Medium	7.71 (4.11–14.47)	Yes
Education	Has incontinence	8.78	0.012*	0.209	Small	-	Yes
Residence	Has incontinence	0.17	0.683	0.029	Small	0.83 (0.44–1.57)	No
Clinical Variables							
Illness duration	Has incontinence	5.40	0.067	0.164	Small	-	No
Antidepressants	Has incontinence	2.28	0.516	0.107	Small	-	No
Mood stabilizers	Has incontinence	3.54	0.316	0.133	Small	-	No
Antipsychotics	Has incontinence	12.55	0.014*	0.250	Small	-	Yes
Symptom Severity Variables							
Solid feces incontinence	Has incontinence	5.45	0.142	0.165	Small	-	No
Liquid feces incontinence	Has incontinence	33.14	< 0.001***	0.407	Medium	-	Yes
Gas incontinence	Has incontinence	180.91	< 0.001***	0.951	Large	-	Yes
Lifestyle changes	Has incontinence	4.33	0.115	0.147	Small	-	No

Notes: Statistical significance: *p < 0.05, **p < 0.01, ***p < 0.001. Effect sizes: Small (0.1–0.3), Medium (0.3–0.5), Large ( > 0.5) based on Cramér’s V. Chi-square test used for all associations; Fisher’s exact test applied where appropriate. Complete inability refers to total loss of fecal control (unablefeces variable). Odds ratios calculated only for 2 × 2 contingency tables with adequate cell frequencies

The contingency analysis revealed several highly significant associations between patient characteristics and fecal incontinence outcomes. Gender emerged as the strongest demographic predictor, with female patients showing a profound association with incontinence (χ^2^ = 42.36, *p* < 0.001, Cramér’s V = 0.460, OR = 7.71), representing a medium-to-large effect size. Educational level also demonstrated a significant but smaller association (χ^2^ = 8.78, *p* = 0.012, Cramér’s V = 0.209). Among clinical variables, antipsychotic medication use showed a significant relationship with incontinence occurrence (χ^2^ = 12.55, *p* = 0.014, Cramér’s V = 0.250). The most striking findings emerged from symptom severity assessments, where gas incontinence showed an exceptionally strong association (χ^2^ = 180.91, *p* < 0.001, Cramér’s V = 0.951), indicating near-perfect correlation, followed by liquid feces incontinence (χ^2^ = 33.14, *p* < 0.001, Cramér’s V = 0.407). Complete loss of bowel control demonstrated perfect associations with all incontinence subtypes (χ^2^ = 200.00, *p* < 0.001, Cramér’s V = 1.000), suggesting these variables may represent different manifestations of the same underlying condition. The strong associations between fecal incontinence symptoms and personal history of urinary incontinence indicate potential shared pathophysiological mechanisms affecting overall continence control in this patient population.RetryClaude can make mistakes. Please double-check responses.

Table 8 provides age-stratified analysis of fecal incontinence prevalence and severity across four age groups in the bipolar disorder cohort.

**Table 8:** Age-stratified analysis of fecal incontinence prevalence and severity in bipolar disorder patients

Age Group (years)	N	Patients with Incontinence (n)	Incontinence Rate (%)	Mean Severity Score ± SD	95% CI for Rate	Risk Category
< 30	56	29	51.79	1.14 ± 2.53	38.5–65.0	Moderate
30–44	97	48	49.48	0.69 ± 0.87	39.4–59.6	Moderate
45–59	45	19	42.22	0.53 ± 0.73	27.7–57.8	Low-Moderate
≥60	2	1	50.00	0.50 ± 0.71	1.3–98.7	Moderate*
Total	200	97	48.50	0.78 ± 1.53	41.4–55.6	-

Age-stratified analysis revealed remarkably consistent fecal incontinence rates across all age groups, with the youngest cohort ( < 30 years) showing the highest prevalence at 51.79% (*n* = 29/56), followed closely by the 30–44 years group at 49.48% (*n* = 48/97), while the 45–59 years group demonstrated the lowest rate at 42.22% (*n* = 19/45). Despite similar prevalence rates, younger patients exhibited significantly higher symptom severity, with mean scores declining progressively from 1.14 ± 2.53 in the < 30 group to 0.53 ± 0.73 in the 45–59 group. The overall incontinence prevalence of 48.50% (97/200 patients) represents a substantially elevated rate compared to community-dwelling adults, where prevalence typically ranges from 2 to 8%. Confidence intervals for individual age groups showed considerable overlap, particularly between the < 30 and 30–44 groups (38.5–65.0% vs 39.4–59.6%), indicating no statistically significant age-related differences in incontinence occurrence. These findings challenge conventional assumptions about age-related incontinence patterns and suggest that psychiatric and pharmacological factors may be the primary determinants of bowel dysfunction in bipolar patients. The consistent high prevalence across all age groups emphasizes the critical need for routine incontinence screening in bipolar disorder management, regardless of patient age.

## Discussion

This cross-sectional study of 200 hospitalized bipolar disorder patients represents the first systematic investigation into fecal incontinence prevalence and associated factors in this population, revealing clinically significant findings regarding bowel dysfunction in psychiatric patients. The analysis demonstrated an exceptionally high overall fecal incontinence prevalence of 48.5% (97/200 patients), substantially exceeding community rates of 2–8%. Notably, incontinence rates remained consistently elevated across all age groups (42–52%), differing from typical age-related patterns observed in general populations. Most importantly, female gender was strongly associated with fecal incontinence (OR = 7.71, 95% CI: 4.11–14.47, *p* < 0.001), with 72% of women experiencing symptoms compared to 25% of men. Psychiatric medication analyses revealed significant heterogeneity within drug classes rather than consistent class-wide associations. These findings suggest that demographic factors, particularly gender and educational level, may be more strongly associated with incontinence outcomes than broad medication categories or disease-related variables. These associations highlight the potential importance of routine incontinence screening in bipolar disorder management, regardless of patient age or current medication regimen.

Our study’s finding of 48.5% fecal incontinence prevalence in hospitalized bipolar disorder patients represents a substantially elevated rate compared to community-based populations, where prevalence typically ranges from 2.0% to 20.7% with a median of 7.7% according to Ng et al.‘s systematic review [11]. This dramatic difference underscores the unique vulnerability of psychiatric populations to bowel dysfunction, with rates approaching those observed in nursing home residents (≈50%) as reported by Nelson [37]. The consistency of high prevalence across all age groups in our study (42–52%) contrasts sharply with the typical age-related progression observed in general populations, where Ng et al. demonstrated increasing prevalence from 5.7% in adults aged 15–34 years to 15.9% in those over 90 years, and Kang et al. [38] found higher rates in Korean adults over 50 years (10.4% vs 4.9%). The most striking finding of our study was the profound gender disparity, with female patients showing 2.88 times higher risk (OR = 7.71) and 72% experiencing symptoms compared to 25% of males. This finding diverges significantly from established literature, where both Ng et al.‘s systematic review and Kang’s Korean population study reported no gender differences in fecal incontinence prevalence. This discrepancy suggests that psychiatric illness and associated treatments may disproportionately affect female bowel function through mechanisms not yet fully understood, potentially involving hormonal interactions, medication metabolism differences, or distinct pathophysiological responses to psychiatric medications. Our analysis of psychiatric medication effects revealed complex, drug-specific patterns rather than consistent class-wide associations. While haloperidol (RR = 1.56) and quetiapine (RR = 1.54) emerged as significant risk factors, olanzapine appeared protective (RR = 0.46). These findings align with emerging evidence of antipsychotic-related bowel dysfunction, as demonstrated in pediatric populations where Zahed et al. [39] found risperidone effective for treating fecal incontinence in children with psychiatric comorbidities, while Aygun [40] reported risperidone-induced fecal incontinence in a 13-year-old patient. The bidirectional effects we observed suggest that different antipsychotics may have opposing impacts on bowel function through varying receptor affinity profiles and gastrointestinal motility effects. The strong association between urinary and fecal incontinence observed in our study population corroborates Nelson’s [11] findings that urinary incontinence represents the greatest risk factor for developing fecal incontinence. Similarly, Demir et al. [41] identified urinary incontinence as a primary risk factor in elderly Turkish outpatients (OR not specified), supporting the concept of shared pathophysiological mechanisms affecting overall continence control. However, our study’s finding that polypharmacy effects were medication-specific rather than dose-dependent differs from Demir’s identification of polypharmacy as a general risk factor, suggesting that drug interactions in psychiatric populations may be more complex than in general elderly populations.

Our investigation represents the first comprehensive systematic evaluation of fecal incontinence in hospitalized bipolar disorder patients, utilizing validated assessment tools (Vaizey and Wexner scoring systems) and employing robust statistical methodology with appropriate non-parametric testing. The study achieved complete data collection without exclusions, ensuring representative findings within the target population. The detailed medication-specific analysis provided unprecedented insights into individual drug effects rather than broad class-wide associations, offering clinically actionable information for medication selection and patient counseling. Several methodological constraints limit the generalizability of our findings. The cross-sectional design precludes establishment of causal relationships between medications and incontinence outcomes, while the hospital-based recruitment may have introduced selection bias toward more severely affected patients. The predominantly Iranian population and single-center design limit external validity to other ethnic groups and healthcare settings. Additionally, the extremely unbalanced medication exposure groups (particularly for antidepressants with only one unexposed patient) restricted comparative analyses and may have influenced risk calculations. The study’s focus on inpatient populations may not reflect outcomes in stable outpatient bipolar disorder patients, potentially overestimating prevalence rates in the broader bipolar population. These findings collectively suggest that fecal incontinence in bipolar disorder patients represents a distinct clinical phenomenon requiring specialized assessment and management approaches, with implications for both psychiatric treatment selection and comprehensive patient care protocols. The extremely unbalanced medication exposure groups represent a statistical limitation. Specifically, only one patient was not receiving antidepressant therapy, and several medication subgroups (lamotrigine: *n* = 13; haloperidol: *n* = 4; pimozide: *n* = 2; carbamazepine: *n* = 2) had insufficient sample sizes to provide stable risk estimates or adequate statistical power. Post-hoc power analysis revealed that comparisons involving fewer than 10 exposed individuals achieved less than 30% power to detect moderate effect sizes (Cohen’s d = 0.5), increasing the risk of both type II errors (failing to detect true associations) and unstable point estimates with wide confidence intervals. This imbalance reflects the clinical reality of inpatient psychiatric care, where polypharmacy is standard practice and monotherapy is rare. However, it limits our ability to isolate the independent effects of individual medications or perform meaningful adjusted analyses controlling for concurrent medication use. The medication-specific risk estimates presented in Table 5, particularly for rarely prescribed agents, should be considered exploratory and hypothesis-generating rather than definitive, requiring validation in larger independent cohorts with more balanced exposure distributions [42, 43].

The present study reveals several significant findings regarding fecal incontinence in hospitalized bipolar disorder patients, with important implications for understanding the complex interplay between psychiatric conditions, pharmacological interventions, and bowel dysfunction. As demonstrated in Table 4, the most striking finding was the pronounced gender-related difference in fecal incontinence severity, with female patients demonstrating significantly higher mean scores compared to males (1.12 ± 1.00 vs 0.44 ± 1.84, *p* < 0.001). This substantial gender disparity, further supported by the risk analysis in Table 5 (OR = 7.71, 95% CI: 4.11–14.47), aligns with previous research establishing associations between psychiatric disorders and incontinence, where hormonal influences and anatomical differences contribute to differential susceptibility patterns [44]. The physiological basis for this gender difference may involve complex interactions between estrogen fluctuations, pelvic floor anatomy, and neurotransmitter systems that regulate both mood and bowel function. Additionally, the correlation between educational attainment and incontinence severity shown in Table 4 (*p* = 0.008) suggests that socioeconomic factors, health literacy, or reporting behaviors may influence symptom recognition and management strategies in this population. The medication-specific effects observed in our risk analysis (Table 5) revealed heterogeneous associations within drug classes. Individual antipsychotics showed markedly different risk profiles, with haloperidol associated with higher risk (RR = 1.56, OR = 3.26) and olanzapine associated with lower risk (RR = 0.46, OR = 0.30). These differential associations may reflect varying receptor binding profiles and anticholinergic properties among antipsychotic medications [15, 45]. Haloperidol’s dopamine D2 receptor blockade and anticholinergic activity may contribute to impaired gastrointestinal motility [46], while olanzapine’s complex receptor profile might involve different mechanisms [47]. Similarly, valproate use was associated with increased incontinence risk (RR = 1.32, OR = 1.63), consistent with prior reports of gastrointestinal effects [48]. The striking 2.88-fold increased risk of fecal incontinence in female bipolar patients suggests complex interactions between hormonal, anatomical, and pharmacological factors. Hormonal fluctuations may influence gastrointestinal motility through effects on smooth muscle contractility and neurotransmitter modulation [49]. Anatomical differences in pelvic floor structure may contribute to increased vulnerability to sphincter dysfunction [50]. Additionally, pharmacokinetic differences between genders, including body composition and hepatic enzyme activity variations, may be associated with differential drug exposure and gastrointestinal effects [51]. However, our cross-sectional design precludes definitive conclusions about causal pathways, and these potential mechanisms require prospective investigation.

The absence of significant differences in incontinence severity between patients with and without broad medication classes (Table 6) reinforces the importance of drug-specific rather than class-wide risk assessments. This finding suggests that the pharmacological heterogeneity within each medication class creates opposing effects that neutralize overall class-level associations. The enteric nervous system, which governs bowel function, contains receptors for dopamine, serotonin, acetylcholine, and histamine—all targets of psychiatric medications—making it particularly susceptible to drug-induced dysfunction [52, 53]. The exceptionally strong associations observed between different incontinence subtypes in Table 7 (gas incontinence: Cramér’s V = 0.951; liquid feces incontinence: Cramér’s V = 0.407) indicate that these symptoms likely represent different manifestations of the same underlying pathophysiological process rather than independent conditions. This supports the concept of a unified continence control system that, when disrupted by psychiatric medications or neurochemical imbalances associated with bipolar disorder, affects multiple aspects of bowel function simultaneously.

The age-stratified analysis (Table 8) revealed an unexpected pattern, with consistently high incontinence rates across all age groups (42–52%) and paradoxically higher symptom severity in younger patients. This finding challenges conventional understanding of age-related incontinence patterns and suggests that psychiatric and pharmacological factors may override typical age-related physiological changes in this population [54]. The overall prevalence of 48.5% presented in Table 8 represents a dramatically elevated rate compared to community-dwelling adults (2–8%), indicating that bipolar disorder and its treatment create substantial bowel dysfunction burden [55]. The physiological explanation for this elevated prevalence likely involves multiple pathways: disrupted autonomic nervous system function inherent to bipolar disorder, medication-induced alterations in neurotransmitter systems governing bowel control, and potential shared genetic or neurobiological vulnerabilities affecting both mood regulation and continence mechanisms. These findings emphasize the critical need for comprehensive assessment protocols that include routine incontinence screening in bipolar disorder management, regardless of patient age, and highlight the importance of individualized medication selection considering both psychiatric efficacy and gastrointestinal safety profiles.

The identification of medication-specific risk profiles in our study necessitates development of comprehensive clinical management protocols for bipolar patients experiencing fecal incontinence. Evidence-based interventions should include systematic medication review with consideration of antipsychotic switching from high-risk agents (haloperidol, quetiapine) to potentially protective alternatives (olanzapine), while maintaining psychiatric stability [56]. Pelvic floor physiotherapy, biofeedback training, and specialized continence programs have demonstrated efficacy in medication-induced bowel dysfunction, with success rates of 60–80% when implemented early [57]. Dietary modifications including fiber supplementation, probiotics, and elimination of trigger foods should be integrated into treatment plans, as gut microbiome alterations associated with both bipolar disorder and psychiatric medications contribute to dysmotility. Routine screening using validated instruments like the Vaizey and Wexner scales should be implemented at baseline and follow-up visits, as patient disclosure rates remain low due to stigma and assumptions about medication necessity.

Several methodological constraints limit the generalizability and causal interpretation of our findings beyond those previously acknowledged. The hospital-based recruitment may have introduced Berkson’s bias, potentially overrepresenting severe cases with multiple comorbidities while underrepresenting stable outpatients who constitute the majority of bipolar disorder patients in clinical practice. The cross-sectional design precluded assessment of temporal relationships between medication initiation and incontinence onset, limiting our ability to establish causality versus correlation in observed associations. Unmeasured confounding variables including dietary habits, physical activity levels, comorbid medical conditions (diabetes, inflammatory bowel disease), and concurrent non-psychiatric medications may have influenced results significantly [58]. Additionally, data on nicotine and caffeine consumption patterns, as well as illicit drug use history, were not systematically collected in this study. These substances are known to affect gastrointestinal motility and may interact with psychiatric medications [59, 60]. Given the relatively high prevalence of substance use in bipolar populations [61], future investigations would benefit from incorporating comprehensive substance use assessments to better clarify the independent contributions of psychiatric medications versus lifestyle factors in bowel dysfunction. The absence of medication dosage, duration of therapy, and adherence data represents a critical limitation, as dose-response relationships are essential for establishing causal inference in pharmacoepidemiological studies. Additionally, cultural factors affecting symptom reporting and the predominantly Iranian population may limit external validity to other ethnic groups with different genetic polymorphisms affecting drug metabolism.

Future investigations should prioritize longitudinal cohort studies following bipolar patients from medication initiation to incontinence development, utilizing electronic health records and patient-reported outcome measures to establish temporal relationships and dose-response patterns. Randomized controlled trials evaluating systematic medication switching protocols in patients with established incontinence could provide crucial evidence for clinical decision-making, comparing psychiatric stability with continence improvement across different antipsychotic and mood stabilizer regimens. Biomarker research investigating genetic polymorphisms in drug-metabolizing enzymes (CYP2D6, CYP3A4) and neurotransmitter receptors could identify patients at highest risk for developing medication-induced bowel dysfunction, enabling personalized medicine approaches. Mechanistic studies utilizing advanced neuroimaging techniques and gut-brain axis biomarkers could elucidate the pathophysiological pathways linking bipolar disorder, psychiatric medications, and continence control. International multicenter collaborations should be established to investigate cross-cultural differences and validate findings across diverse populations with varying genetic backgrounds and healthcare systems.

Implementation of systematic screening protocols requires development of brief, validated assessment tools suitable for routine psychiatric practice, incorporating digital health platforms and patient-reported outcome measures that can be completed remotely. Clinical decision support systems integrated into electronic health records should provide real-time alerts for high-risk medication combinations and prompt consideration of alternative therapeutic options when prescribing patterns suggest increased incontinence risk. Multidisciplinary care teams including psychiatrists, gastroenterologists, and specialized continence nurses should be established in major psychiatric centers to provide comprehensive assessment and management. Training programs for mental health professionals should incorporate continence assessment skills and knowledge of medication-specific gastrointestinal effects, as current psychiatric education inadequately addresses these complications. Patient education materials and shared decision-making tools should be developed to facilitate informed discussions about medication risks and benefits, empowering patients to participate actively in treatment planning.

The complex interplay between demographic, clinical, and pharmacological factors observed in our study suggests that traditional single-variable analyses may inadequately capture the multifactorial nature of medication-induced incontinence. Advanced statistical modeling techniques including machine learning algorithms and network analysis could identify previously unrecognized interaction patterns between age, gender, medication combinations, and genetic factors [62]. Polypharmacy effects, particularly common in bipolar disorder management where patients often receive multiple psychiatric medications simultaneously, may produce synergistic or antagonistic effects on bowel function that cannot be predicted from individual drug profiles [63]. Gene-environment interactions involving cytochrome P450 polymorphisms, dietary factors, and medication adherence patterns may explain the substantial individual variability observed in our cohort, suggesting that personalized risk prediction models could improve clinical outcomes. The potential for epigenetic modifications induced by chronic psychiatric medication exposure to alter gastrointestinal function represents an emerging area requiring investigation, as these changes may persist even after medication discontinuation and explain some of the irreversible bowel dysfunction observed in long-term psychiatric patients.

## Conclusion

Based on the comprehensive analysis of 200 hospitalized bipolar disorder patients, this study reveals a concerning prevalence of fecal incontinence (48.5%) that substantially exceeds community rates, with female gender emerging as the most significant risk factor (OR = 7.71, 95% CI: 4.11–14.47). The findings demonstrate that demographic factors, particularly gender and educational attainment, are more predictive of incontinence outcomes than broad medication classes, while individual psychiatric medications within the same therapeutic class exhibit markedly different risk profiles. Notably, haloperidol and quetiapine increased incontinence risk, whereas olanzapine appeared protective, indicating that medication-specific rather than class-wide assessments are essential for clinical decision-making. The consistent high prevalence across all age groups (42–52%) challenges conventional age-related incontinence patterns and suggests that psychiatric and pharmacological factors override typical physiological aging effects in this vulnerable population. These findings underscore the urgent need for systematic integration of continence assessment into routine bipolar disorder management, regardless of patient age or current medication regimen. The profound gender disparity observed necessitates development of sex-specific screening protocols and consideration of hormonal, anatomical, and pharmacokinetic factors when selecting psychiatric medications for female patients. Future research should prioritize longitudinal studies to establish causal relationships between specific medications and incontinence development, while clinical practice should implement validated screening tools and multidisciplinary care approaches incorporating psychiatrists, gastroenterologists, and specialized continence specialists. The substantial burden of bowel dysfunction in bipolar patients represents a previously underrecognized complication that significantly impacts quality of life and may influence treatment adherence, highlighting the critical importance of comprehensive, patient-centered care that addresses both psychiatric symptoms and medication-related adverse effects through evidence-based management strategies.

## Data Availability

The datasets used and/or analyzed during the current study are available from the corresponding author on reasonable request.
